# Olikomycin A Disrupts Gram‐Positive Bacterial Membranes Through Calcium‐Dependent Binding to Phosphatidylglycerol

**DOI:** 10.1002/ardp.70318

**Published:** 2026-08-17

**Authors:** Luisa Munz, Olga Makshakova, Sara Marchi, Winfried Römer, Andreas Bechthold

**Affiliations:** ^1^ Pharmaceutical Biology and Biotechnology Institute for Pharmaceutical Sciences University of Freiburg Freiburg Baden‐Württemberg Germany; ^2^ Synthetic Biology of Signaling Processes, Faculty of Biology University of Freiburg Freiburg Baden‐Württemberg Germany; ^3^ CIBSS – Centre for Integrative Biological Signaling Studies University of Freiburg Freiburg Baden‐Württemberg Germany

**Keywords:** antimicrobial mechanism, calcium‐dependent antibiotics, membrane disruption, olikomycin A, phosphatidylglycerol

## Abstract

Calcium‐dependent antibiotics (CDAs) represent an important class of antimicrobial agents active against Gram‐positive pathogens. Olikomycin A, produced by *Streptomyces ghanaensis* Δ*wblA*, displays potent activity against multidrug‐resistant bacteria; however, its molecular mode of action remained unclear. In this study, we investigated the interaction of olikomycin A with bacterial membrane lipids. Phospholipid antagonization assays demonstrated a calcium‐dependent interaction with phosphatidylglycerol (PG), a major anionic phospholipid of Gram‐positive membranes. Cryo‐electron microscopy and fluorescence microscopy using model membranes revealed rapid membrane disruption induced by olikomycin A, resulting in vesicle deformation and fragmentation. Compared with the clinically used lipopeptide daptomycin, olikomycin A caused markedly faster and more extensive membrane damage. Furthermore, treatment of *Staphylococcus aureus* with olikomycin A in the presence of calcium resulted in rapid cellular aggregation consistent with severe membrane perturbation. These findings indicate that olikomycin A targets bacterial membranes via calcium‐dependent binding to PG and disrupts membrane integrity through a mechanism distinct from daptomycin.

## Graphical abstract

Olikomycin A, a calcium‐dependent lipopeptide antibiotic, targets Gram‐positive bacteria by binding to phosphatidylglycerol and rapidly disrupting membrane integrity. Unlike daptomycin, it induces extensive membrane remodeling, vesicle deformation, and fragmentation. This aggressive mechanism leads to rapid membrane collapse and cellular aggregation, highlighting olikomycin A as a promising candidate against multidrug‐resistant pathogens. Created in BioRender. Marchi, S. (2026) https://BioRender.com/2jmbr3y

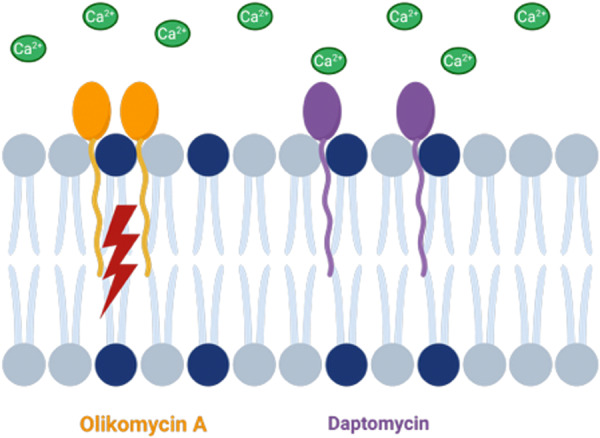

## 1 Introduction

**Figure 1:**
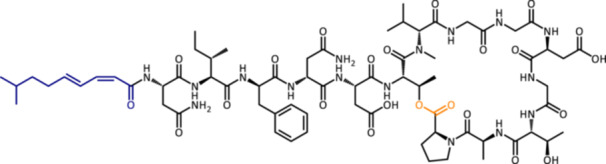
Structure of olikomycin A, the N‐terminal lipid chain in shown in blue, the cyclisation site via an ester bond in shown in orange.

The increasing prevalence of multidrug‐resistant Gram‐positive pathogens [1] highlights the urgent need for antibiotics with novel mechanisms of action. Calcium‐dependent antibiotics (CDAs) constitute a family of cyclic lipopeptides that require calcium ions for antibacterial activity and typically target bacterial membranes. The clinically used lipopeptide daptomycin represents the best‐known member of this group and has been proposed to bind to phosphatidylglycerol (PG) and form membrane pores [2, 3, 4], although alternative mechanisms have also been suggested [5, 6]. Olikomycin A is a CDA produced by *Streptomyces ghanaensis* [7] (Figure 1). Previous studies demonstrated potent antibacterial activity against Gram‐positive pathogens, but the underlying molecular mechanism has remained largely unknown. In the present study, we investigated whether olikomycin A interacts with bacterial membrane phospholipids and how this interaction affects membrane integrity.

## 2 Results and Discussion

Structurally, olikomycin A consists of a lipid tail attached to a cyclic peptide core, a characteristic feature of calcium‐dependent lipopeptide antibiotics. Although CDAs are generally not known to inhibit peptidoglycan biosynthesis directly, several members of this family exert their antibacterial activity through membrane disruption. To examine whether olikomycin A interacts with bacterial membrane phospholipids, we performed phospholipid antagonization assays using the zwitterionic phospholipid 1,2‐dioleoyl‐sn‐glycero‐3‐phosphocholine (DOPC) and anionic phospholipids including 1,2‐dioleoyl‐sn‐glycero‐3‐phosphoglycerol (DOPG). Minimum inhibitory concentrations (MICs) were determined against *Staphylococcus warneri* DSM 2361 in the presence or absence of calcium ions (Table 1). Daptomycin served as a positive control due to its known interaction with PG, whereas vancomycin, which targets lipid II rather than membrane phospholipids, was used as a negative control [8].

**Table 1:** Results of MIC tests against *Staphylococcus warneri* DSM 2361 after incubation with calcium and/or the phospholipids DOPC, DOPG, Cardiolipin, and POPS.

	MIC				
	Ca^2+^	DOPC + Ca^2+^	DOPG + Ca^2+^	POPS + Ca^2+^	Cardiolipin + Ca^2+^
Olikomycin A	4	4	> 64	8	> 64
Daptomycin	< 0.125	< 0.125	> 64	< 0.125	4
Vancomycin	2	2	2	2	2

Abbreviations: DOPC, 1,2‐dioleoyl‐sn‐glycero‐3‐phosphocholine; DOPG, 1,2‐dioleoyl‐sn‐glycero‐3‐phosphoglycerol; MIC, minimum inhibitory concentrations; POPS, 1‐palmitoyl‐2‐oleoyl‐sn‐glycero‐3‐phospho‐l‐serine.

In the presence of DOPG and calcium, the MIC values of both olikomycin A and daptomycin increased markedly (> 64 µg mL^−1^), whereas vancomycin activity remained unaffected. Additionally, the MIC of olikomycin A increased in the presence of Cardiolipin to > 64 µg mL^−1^, whereas the MIC of daptomycin increased only slightly to 4 µg mL^−1^. The presence of 1‐palmitoyl‐2‐oleoyl‐sn‐glycero‐3‐phospho‐l‐serine (POPS) and calcium changed the MIC of olikomycin A to 8 µg mL^−1^ and the MIC of daptomycin stayed at < 0.125 µg mL^−1^. These findings indicate that olikomycin A, similar to daptomycin, interacts specifically with anionic phospholipids such as PG and partially Cardiolipin. The difference in MIC values suggest a weaker interaction for daptomycin and Cardiolipin and no interaction for daptomycin and POPS. The interaction of olikomycin A and POPS points to an attenuated interaction compared to the other anionic phospholipids.

**Figure 2:**
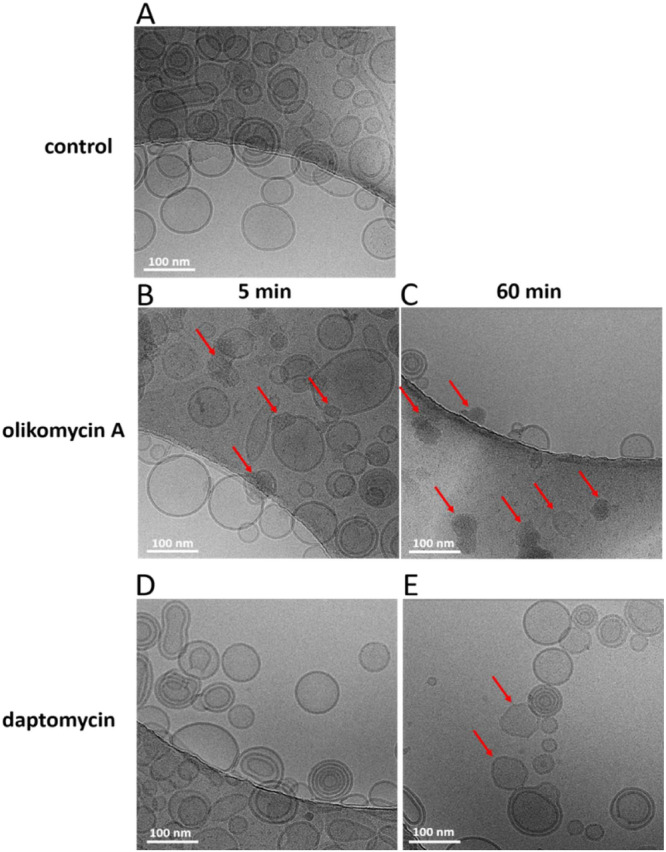
CryoEM images of LUVs incubated with calcium (A), olikomycin A and calcium for 5 min (B) and 60 min (C), daptomycin and calcium for 5 min (D) and 60 min (E). The red arrows point to damaged LUVs showing the effect of the antibiotics. LUVs, large unilamellar vesicles.

**Figure 3:**
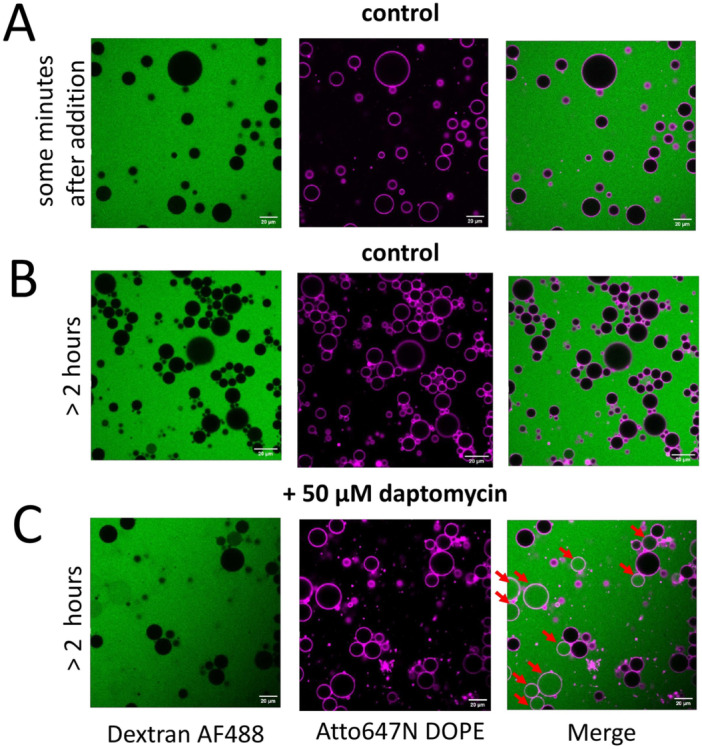
Effects of daptomycin on the integrity and morphology of giant unilamellar vesicles. GUVs were incubated with fluorescent Dextran‐Alexa Fluor 488 (3 kDa) at room temperature for some minutes (A) and more than 2 h (B) and in addition with 50 µM daptomycin (C). The intensities were slightly adapted. The red arrows point to leaky GUVs showing the effect of the antibiotics. Scale bars: 20 µm. GUVs, giant unilamellar vesicles.

**Figure 4:**
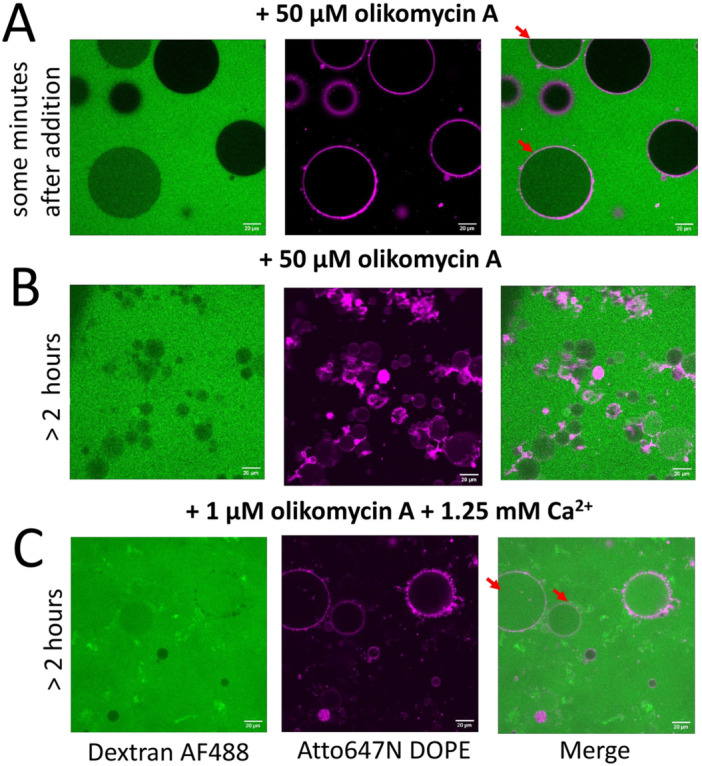
Effects of olikomycin A on the integrity and morphology of giant unilamellar vesicles. GUVs were incubated with fluorescent Dextran‐Alexa Fluor 488 (3 kDa) at room temperature with 50 µm of olikomycin A for some minutes (A) and more than 2 h (B), and with 1 µM olikomycin A and 1.25 mM calcium (C). The intensities were slightly adapted. The red arrows point to leaky GUVs showing the effect of the antibiotics. Scale bars: 20 µm. GUVs, giant unilamellar vesicles.

To further investigate the membrane‐active properties of olikomycin A, we performed cryo‐electron microscopy (cryo‐EM) experiments using large unilamellar vesicles (LUVs). Untreated vesicles showed intact membranes with no detectable structural alterations (Figure 2A). In contrast, vesicles exposed to olikomycin A displayed clear membrane damage within minutes (Figure 2B), progressing to extensive vesicle destruction and formation of electron‐dense debris after prolonged incubation (Figure 2C). Under identical conditions, daptomycin caused only minor vesicle deformation (Figure 2D,E). Membrane permeabilization was further analyzed using confocal fluorescence microscopy with giant unilamellar vesicles (GUVs) [9]. Control vesicles maintained their spherical morphology and excluded fluorescent dextran (Figure 3A,B). Treatment with daptomycin resulted in gradual dextran influx (Figure 3C) while largely preserving vesicle structure, which may be indicative of pore formation or another membrane‐permeabilizing mechanism. In contrast, olikomycin A induced rapid dextran entry accompanied by pronounced morphological changes, including membrane deformation and vesicle budding (Figure 4A,B). Notably, these effects were also observed at lower concentrations of olikomycin A in the presence of calcium ions.

**Figure 5:**
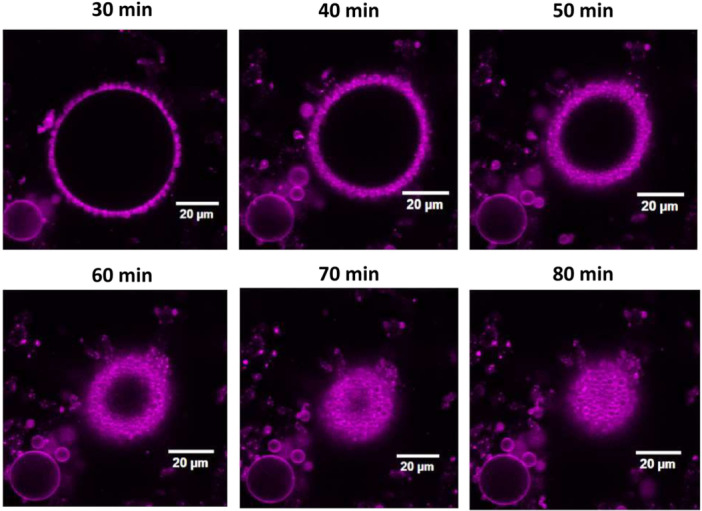
Time series illustrating the effects of 1 µM olikomycin A in combination with 1.25 mM calcium on membrane morphology. The intensities were slightly adapted. Scale bars: 20 µm.

Time‐lapse imaging revealed dynamic reorganization of the membrane, including redistribution of fluorescent lipids and formation of smaller vesicular structures (Figure 5). These observations indicate that olikomycin A destabilizes membrane architecture rather than forming discrete pores.

**Figure 6:**
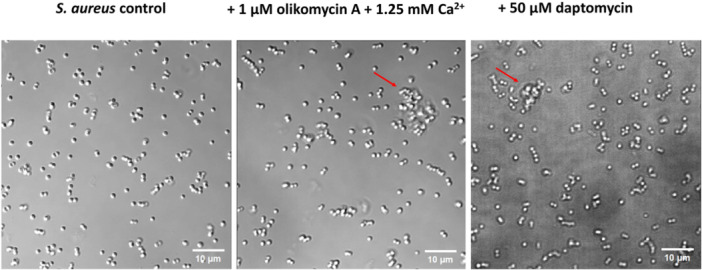
Interacting of olikomycin A and daptomycin with *Staphylococcus aureus*. Clots formation (pointed by a red arrow) was observed in the presence of 1 µM olikomycin A and 1.25 mM calcium and 50 µM daptomycin.

Finally, the activity of olikomycin A was evaluated in *Staphylococcus aureus*. Exposure to 1 µM olikomycin A in the presence of calcium ions as well as 50 µM daptomycin resulted in rapid cellular aggregation, consistent with severe membrane damage (Figure 6).

Taken together, our results demonstrate that olikomycin A binds to the anionic phospholipid PG and rapidly disrupts membrane integrity. In contrast to daptomycin, for In contrast to daptomycin, for which one proposed mode of action involves forming defined pores [10, 11], olikomycin A induces extensive membrane remodeling and vesicle fragmentation, suggesting a distinct and potentially more aggressive mechanism of membrane disruption.

## 3 Experimental

### 3.1 General Experimental Procedures

Water used for chromatographic separations was purified to a resistivity of 18.2 MΩ·cm at 25°C using a Milli‐Q purification system. Acetonitrile (ACN) and formic acid were obtained in HPLC or LC–MS grade. All other solvents and reagents were purchased from commercial suppliers and used without further purification unless otherwise stated.

### 3.2 Isolation of Olikomycin A

Olikomycin A was obtained from cultures of *Streptomyces ghanaensis* Δ*wblA*. Natural product production was initiated by inoculating a stock culture into 20 mL tryptic soy broth (TSB). The preculture was incubated at 28°C with shaking at 180 rpm for 24 h. Subsequently, 1 mL of the preculture was transferred to production medium and cultivated for 5 days at 28°C under aerobic conditions. For extraction, the culture broth was centrifuged (20 min, 8000 rpm, room temperature) to separate the biomass from the supernatant. The supernatant was filtered and transferred to a separatory funnel. An equal volume of n‐butanol was added and the biphasic mixture was shaken for 30 min to ensure efficient extraction of secondary metabolites. In some cases, an emulsion layer formed between the aqueous and organic phases; this was resolved by addition of 5–10 mL acetone and, if necessary, additional n‐butanol. The organic phase was separated and concentrated to dryness under reduced pressure using a rotary evaporator. The resulting crude extract was stored at −20°C until further purification. Purification of olikomycin A was achieved by preparative HPLC using a C18 column from XBridge™. As solvent we used acetonitrile and water. Fractions containing the target compound were identified by LC–MS analysis and pooled to yield purified olikomycin A.

### 3.3 Determination of Minimum Inhibitory Concentrations (MIC)

MICs were determined using a broth microdilution assay according to EUCAST guidelines, with modifications. Tests were performed in 96‐well microtiter plates using Luria–Bertani (LB) medium, since precipitation of calcium ions occurred when the standard medium (cation‐adjusted Mueller–Hinton broth) was used. Bacterial strains were precultured overnight and diluted the following morning. Cultures were adjusted to OD600 = 0.1 and subsequently diluted 80–100 fold, depending on the strain, to obtain a final inoculum of 5 × 10^5^ CFU mL^−1^. Serial dilutions of olikomycin A or control antibiotics were prepared in LB medium. 50 µL of each antibiotic dilution were added to the wells followed by 50 µL of bacterial suspension.

Each plate included growth control (bacteria without antibiotic) and Sterility control (medium without bacteria). Plates were incubated under appropriate growth conditions and MIC values were determined as the lowest compound concentration that prevented visible bacterial growth.

### 3.4 Phospholipid Antagonization Assay

To investigate the interaction of olikomycin A with membrane phospholipids, phospholipid antagonization assays were performed using the phospholipids DOPC, DOPG, 1′,3′‐bis[1,2‐dioleoyl‐sn‐glycero‐3‐phospho]‐glycerol (Cardiolipin) and 1‐palmitoyl‐2‐oleoyl‐sn‐glycero‐3‐phospho‐l‐serine (POPS). Olikomycin A was incubated with the phospholipids in the presence or absence of Ca^2+^ ions, followed by determination of MIC values against *S. warneri* DSM 2361 using the microdilution method described above. Daptomycin served as a positive control due to its known interaction with phosphatidylglycerol, whereas vancomycin was used as a negative control.

### 3.5 Preparation of Large Unilamellar Vesicles (LUVs)

Large unilamellar vesicles (LUVs) were prepared using standard lipid film hydration and extrusion procedures. Lipid mixtures were hydrated to obtain a total lipid concentration of 3.5 mM. The vesicle suspensions were incubated with olikomycin A or daptomycin in the presence of Ca^2+^ ions prior to structural analysis by cryo‐electron microscopy. In brief a lipid film was prepared from a DOPC/DOPG mixture (3:1) in the presence of HEPES buffer. To promote liposome formation, the dispersion was subjected to three freeze–thaw cycles. Extrusion was carried out 15 times through two stacked 80 nm polycarbonate membranes in a thermobarrel extruder. The extrusion was performed at 25°C using pressurized nitrogen at approximately 20 bar. The final liposome size was determined by dynamic light scattering (DLS). The aim was to produce LUVs with a mean diameter of approximately 100 nm. The concentration was determined using a Bartlett assay [12].

### 3.6 Cryo‐Electron Microscopy (cryo‐EM)

Membrane integrity of LUVs was analyzed by cryo‐EM. Krios G4 300 kV Transmission Electron Microscope (TEM) from Thermofisher was used. Vesicle samples were incubated with the antibiotics for defined time intervals before vitrification. Cryo‐EM imaging allowed visualization of membrane morphology and structural alterations induced by antibiotic treatment, including vesicle deformation, membrane rupture, and formation of electron‐dense membrane fragments.

### 3.7 Preparation of GUVs

GUVs were prepared by the electroformation method as described [13] using lipid mixtures consisting of POPC/POPG (75:25 mol%). In brief, lipid mixtures dissolved in chloroform at a final concentration of 0.5 mg/mL were deposited onto indium tin oxide (ITO)‐coated glass slides. The solvent was removed by drying the slides under vacuum for at least 1 h or overnight. Subsequently, two ITO slides were assembled to form a chamber that was filled with a sucrose solution adjusted to match the osmolarity of PBS for imaging of GUVs. An alternating electric field with a strength of 1 V/mm was then applied for 2.5 h at room temperature to promote vesicle formation. The resulting GUVs were afterwards transferred to manually assembled observation chambers as described previously. For fluorescence visualization, membranes were labeled with DOPE‐Atto647N, while membrane permeability was evaluated using Dextran‐Alexa Fluor 488 (3 kDa) as a soluble fluorescent tracer.

### 3.8 Fluorescence Microscopy

Membrane permeabilization and vesicle morphology were analyzed using confocal fluorescence microscopy (NIKON A1R confocal microscope, 60 × oil objective, NA = 1.49, used laser wavelengths: 405 nm, 488 nm, and 647 nm). GUVs were incubated with olikomycin A or daptomycin at the indicated concentrations in the absence or presence of calcium ions. Dextran influx into the vesicle lumen was monitored as an indicator of membrane permeability, while morphological changes such as vesicle deformation, budding, or fragmentation were recorded during time‐lapse imaging.

### 3.9 Bacterial Aggregation Assay

To evaluate cellular effects of olikomycin A, *Staphylococcus aureus* cells were incubated with 1 µM olikomycin A and 50 µM daptomycin respectively in the presence of 1.25 mM Ca^2+^. The images were taken after 30 min of incubation.

Cells were examined microscopically for aggregation phenomena indicative of severe membrane perturbation. Aggregation and clot‐like structures were compared with control treatments.

## Conflicts of Interest

The authors declare no conflict of interest.

## Data Availability

The data that support the findings of this study are available from the corresponding author upon reasonable request.
